# Electrospun PLA/PVP K90 Biphasic-Release Sublingual Film for Motion Sickness Treatment

**DOI:** 10.3390/biom16030363

**Published:** 2026-02-28

**Authors:** Wenwen Zhang, Qilin Wang, Wei Yi, Hongxi Wang, Deng-Guang Yu, Tao Yi

**Affiliations:** 1 School of Materials and Chemistry, University of Shanghai for Science and Technology, Shanghai 200093, China; 233393151@st.usst.edu.cn (W.Z.); 243403174@st.usst.edu.cn (Q.W.); 2 FORYOU Mechatronics (Shanghai) Ltd., Shanghai 201114, China; lily@ablpropharma.com (W.Y.); wang@i-foryou.com.cn (H.W.); 3 Faculty of Health Sciences and Sports, Macao Polytechnic University, Macau 999078, China

**Keywords:** electrospinning, core–sheath structure, biphasic release, sublingual membrane, motion sickness

## Abstract

To overcome the limitations of traditional motion sickness medications—slow onset of action, short duration of efficacy, and poor patient compliance—this study employed coaxial electrospinning technology. Poly(lactic acid) (PLA) and polyvinylpyrrolidone K90 (PVP K90) were used as composite carrier materials. The sheath layer is composed of highly hydrophilic PVP K90, loaded with the antihistamine diphenhydramine (DPH). The core layer, composed of biodegradable PLA with excellent sustained-release properties, carries the anticholinergic drug scopolamine hydrobromide (SH). This core–sheath nanostructured nanofiber sublingual film delivers dual anti-motion sickness drugs. A series of characterization tests revealed that the sublingual membrane exhibits a linear morphology with a distinct core–shell nanostructure. The drugs DPH and SH are distributed in an amorphous state within the sheath and core layers, respectively. Wetting performance tests indicate that the membrane’s wettability falls between those of monofilament membranes. In vitro drug release experiments revealed that DPH exhibited a “rapid onset + sustained release” biphasic profile, with cumulative release reaching 60% within 2 h and approaching complete release by 10 h, primarily via Fickian diffusion (*n* = 0.30). SH exhibited prolonged sustained release, approaching complete release at 12 h via non-Fickian diffusion (*n* = 0.55). Cytotoxicity and vital/necrotic staining experiments mutually corroborated that cell viability remained above 80%, further validating the safety and efficacy of PLA/PVP as a combined drug delivery carrier. This study provides a novel delivery system for motion sickness treatment, offering significant theoretical value and broad clinical application prospects.

## 1. Introduction

Motion sickness (MS), also known as motion sickness, seasickness, or diseases caused by swaying, bumping, rotation, accelerated movement, etc., caused by various reasons, is a visceral dysfunction syndrome caused by the information conflict between human vision, the vestibular system, and proprioception, and mainly clinically manifests as paleness, cold sweat, salivation, apathy, abdominal discomfort, dizziness, nausea, vomiting, and other reactions [1,2,3,4,5,6,7,8]. Approximately 30~70% of the world’s population suffers from motion sickness, which is widely occurring in aviation, navigation, and road traffic [6,9], and the incidence of mental illness in military training (carrier-based aircraft pilots, ship personnel) and space missions [9,10], as well as in emerging fields such as virtual display (VR) experience, has also shown a significant upward trend, seriously reducing the physiological comfort and task performance efficiency of the population. Relevant epidemiological investigations have found that about 80% of the population will have varying degrees of motion sickness under specific stimulus conditions [5], of which 20~30%, will develop moderate to severe symptoms [11], and convenient and effective immediate intervention is urgently needed.

The pathogenesis of motion sickness is closely related to vestibulo-autonomic reflex disorders and abnormal activation of central cholinergic and histaminergic pathways. At present, the prevention and treatment of motion sickness mainly include behavioral interventions (such as choosing a front seat and fixed sight), physical therapy (such as auricular pressures), and medication [12]. Among them, drug treatment is the most direct means of response, but there are many limitations in the current clinical drugs and dosage forms used for the treatment of motion sickness, and there is an urgent need for immediate administration preparations with rapid onset and low adverse reactions. Although traditional oral preparations such as difenidol hydrochloride tablets have a fast onset of action, they require drinking water and swallowing, which means that they struggle to meet the emergency medication needs of non-drinking-water scenarios such as car rides and boat rides, and the drug has a short half-life, requiring repeated administration every 6~8 h, resulting in poor compliance [13]. Scopolamine hydrobromide transdermal patch is a commonly used dosage form for long-acting treatment, which can achieve 72 h of continuous release, but its onset lags and it needs to be applied to the skin behind the ear 4~6 h in advance, meaning that it cannot cope with sudden acute motion sickness. It is also prone to local irritation reactions such as skin itching and erythema, limiting its application in sensitive groups [3]. Nasal sprays or intravenous injections are highly invasive, and patients have poor compliance. In addition, there are often shortcomings in the efficacy of single-drug therapy. Although antihistamines can quickly relieve dizziness and nausea, they are accompanied by obvious drowsiness side effects, and anticholinergics have outstanding long-term effects but a slow onset, and it is difficult to fulfil the clinical needs of “fast-acting” and “long-acting” when medication is used alone.

As an anticholinergic drug, scopolamine is a tropane alkaloid isolated from the nightshade plant, which structurally mimics the human neurotransmitter acetylcholine and acts as an M (M1, M2, M3, M4, M5) choline receptor antagonist, which can target and block the central cholinergic signal in the chemoreceptor zone of the vestibular nucleus and medulla oblongata and, at the same time, antagonize peripheral M receptors to relieve secondary symptoms such as gastrointestinal spasm, as well as being a core anti-motion drug [3]. At the same time, diphenhydramine, as an antihistamine drug, inhibits vestibular neuron excitability by blocking H1 histamine receptors and has both mild anticholinergic and mild sedative effects, which can help enhance the anti-halo effect [14]. The combination of the two can simultaneously block cholinergic and histaminergic dual pathways, covering the entire symptom chain of dizziness, nausea, and vomiting, and the combined treatment dose can significantly reduce adverse reactions, such as dry mouth and drowsiness, caused by a single drug. However, due to the relatively slow onset of oral administration, it usually takes about 30~60 min to reach the peak, and the action time is short. Therefore, there is a need to develop a drug carrier that can disintegrate drugs quickly, has high bioavailability, and can be released continuously for a long time.

As a non-invasive route of administration, oral mucosal drug administration has the advantages of large mucosal surface area, abundant blood vessels, strong permeability (especially sublingual and buccal mucosa), and avoidance of liver first-pass effects, and has been widely used in the research and development of immediate-release formulations [15,16,17,18,19]. Sublingual mucosal administration allows for rapid drug absorption through non-keratinized epithelium and a dense capillary network, entering the systemic circulation directly and bypassing the liver’s first-pass effect, significantly improving bioavailability. At the same time, it offers clinical advantages such as a quick onset of action (1–5 min), good patient compliance, and the ability to stop administration at any time, making it particularly suitable for drugs that require rapid onset or are significantly affected by the first-pass effect [19,20,21,22,23,24]. As a new nanofiber preparation technology, electrospinning technology can prepare nanofiber membranes [25,26,27,28,29,30,31,32] with a high specific surface area and high porosity by regulating the spinning parameters [33,34,35,36,37], and its fiber structure can closely adhere to the sublingual mucosa. At the same time, the electrospinning process can effectively transform crystalline drugs into amorphous solid dispersions (ASDs) in polymer nanofibers, while the rapid evaporation of the solvent prevents drug recrystallization, resulting in molecular-level dispersion, which is beneficial for improving solubility and bioavailability [16,38,39,40,41,42]. Polylactic acid (PLA) is a polyester material with good biocompatibility and degradability, and its hydrophobic properties can confer certain mechanical strength to the fiber membrane [43]. Polyvinylpyrrolidone K90 (PVP K90) is a water-soluble polymer material with excellent film-forming and solubilization properties [36,44], and the compounding of the two can take into account the mechanical properties and drug release properties of the fiber membrane [45]. The PVP K90-loaded DPH outer layer rapidly hydrates to form a high-viscosity mucoadhesive gel layer, prolonging retention time through mucosal adhesion. The PLA core layer acts as an insoluble matrix, maintaining structural integrity to control the sustained release of SH. The design of a coaxial electrospinning-derived core–sheath structure [46,47,48] provides technical support for the construction of biphasic release drug delivery systems [49,50,51,52].

**Figure 1:**
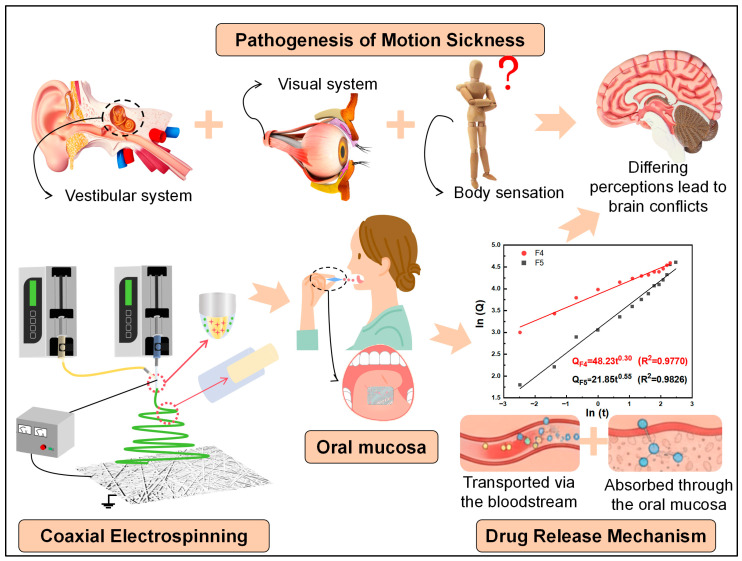
Diagram of the research approach.

Based on this, in this study, coaxial electrospinning technology was used to construct a dual-drug-loaded core–sheath-structure nanofiber biphasic release sublingual membrane [53], and the two anti-halo drugs were innovatively regulated by layered loading and drug release. The sheath layer was made of highly hydrophilic PVP K90 as the carrier material and loaded with the antihistamine diphenhydramine. The core layer was made of biodegradable PLA with excellent sustained-release properties as a carrier, loaded with the anticholinergic drug scopolamine hydrobromide. This study is expected to overcome the limitations of traditional motion sickness drug delivery and provide a novel sublingual film with rapid onset, high bioavailability, and good patient compliance for clinical use, possessing significant theoretical value and clinical application prospects. Figure 1 shows a schematic of this study to facilitate understanding.

## 2. Materials and Methods

### 2.1. Materials

Polyvinylpyrrolidone (PVP KP0, CAS: 9003-39-8) was purchased from Sigma-Aldrich Trading Co., Ltd. (Shanghai, China); polylactic acid (PLA, CAS: 26100-51-6, particle size: 3 mm, Mw: 80,000) was purchased from Macklin Biochemical technology Co., Ltd. (Shanghai, China); Scopolamine Hydrobromide (SH, CAS: 114-49-8) was purchased from Hansuyuan Biotechnology Co., Ltd. (Xi’an, China); Diphenhydramine Hydrochloride (DPH, CAS: 58-73-1, TCLD4744, 98.0% (GC&T)) was purchased from Sinopharm Chemical Reagent Co., Ltd. (Shanghai, China); Trifluoroethanol and Methylene Blue. All chemical reagents were analytical grade, and deionized water was used for experimental purposes. L929 cell line (mouse fibroblast cell line) used in this study was purchased from Procell Life Science & Technology Co., Ltd. (Wuhan, China). MEM culture medium, fetal bovine serum, PBS, trypsin, penicillin–streptomycin solution (100X), and CCK8 were provided by Science Compass (Hangzhou Yanqu Information Technology Co., Ltd., Hangzhou, China).

### 2.2. Preparation of Electrospun Nanofiber Membranes

The electrospinning apparatus used in this experiment was self-assembled and comprised four main components: a high-voltage power supply, two syringe pumps, a custom-made coaxial spinning head, and an aluminum foil fiber collector. Through preliminary experimentation, the working fluid and spinning parameters were finalized as follows: The sheath solution comprised 12% (*w*/*v*) PVP K90 and 15% (*w*/*w*) DPH, with a solvent mixture of TFEA and deionized water at a volume ratio of 95:5. The solution preparation involved dissolving 1.2 g of PVP K90 in 9.5 mL TFEA and 0.5 mL deionized water (total solvent volume: 10 mL) at 500 rpm for 24 h at room temperature. After dissolution, 180 μL DPH was added, and stirring continued for 6 h under light-shielded conditions. The core layer solution consists of 22% (*w*/*v*) PLA and 5% (*w*/*w*) SH in TFEA. The specific steps for solution preparation are as follows: 2.2 g of PLA is added to 10 mL of trifluoroethanol solvent. The mixture is heated at 60 °C in an oil bath at 500 rpm for 24 h. (Heating promotes PLA dissolution while preventing solvent evaporation due to high temperatures.) After complete dissolution, 0.11 g of SH is added to the solution and stirring continues at room temperature for 6 h.

Samples F1 and F2 were prepared using single-fluid electrospinning technology. Core–sheath nanofibers (samples F3, F4, F5, and F86) were produced via coaxial electrospinning. Detailed experimental parameters are listed in Table 1.

Environmental conditions: temperature 22 ± 3 °C; relative humidity 40 ± 5%.

### 2.3. Characterization of Physical Properties

#### 2.3.1. Morphology

The morphology of electrospun nanofibers, including uniaxial nanofibers and core–sheath nanofibers, was evaluated using a scanning electron microscope (SEM, Quanta FEG450, Hillsboro, OR, USA). For sampling, a small piece of the fiber membrane was cut and fixed to the sample stage using double-sided conductive adhesive before placing the sample into the vacuum chamber. Gold sputtering was performed prior to evaluation. Subsequently, the average diameter of nanofibers at approximately 100 locations was estimated from SEM images using ImageJ software V1.8.0 (NIH, Bethesda, MD, USA).

#### 2.3.2. Internal Structure

The internal structure of uniaxial and core–sheath nanofibers was evaluated using transmission electron microscopy (TEM, JEM2200F, JEOL, Akishima, Japan). Samples were prepared by placing a 200-mesh copper screen over the collector for less than 10 s during spinning, followed by placing the collected nanofibers into the vacuum chamber for examination.

#### 2.3.3. Physical State

Testing was performed using an X-ray powder diffractometer (XRD, Bruker-AXS, Karlsruhe, Germany). All raw materials (PVP, PLA, DPH, and SH) and electrospun nanofiber membranes F1–F8 were measured in the 2θ angle range of 10–70°. The applied voltage and operating current were 40 kV and 30 mA, respectively.

#### 2.3.4. Compatibility

Fourier transform infrared (FTIR) analysis was performed using a PerkinElmer FTIR spectrometer (Spectrum 100, Billerica, MA, USA) to investigate compatibility between the polymer carrier and the active pharmaceutical ingredient. Samples were scanned at 500–4000 cm^−1^ with a resolution of 2 cm^−1^. For powdered solid materials, 0.2 g of potassium bromide powder was ground with approximately 10 mg of sample. The mixture was compressed into solid pellets, which were then placed in the instrument for scanning. For liquid drug samples, a drop of liquid was applied to a blank potassium bromide pellet for measurement. For nanofiber membranes, a small piece of the membrane was cut and placed on the sample for scanning.

### 2.4. Functional Performance Evaluation

#### 2.4.1. Mechanical Properties Testing

Standard dumbbell-shaped molds were used to prepare test strips with a central width of 6 mm from nanofiber membranes F1–F8. A micrometer was employed to measure thickness at three points along the length, with the average value recorded as the sample thickness. Tensile testing of the nanofiber membranes was conducted using a 2.5 kN universal testing machine at a strain rate of 2.5 mm/min. The tensile strength and elongation at break were calculated for each sample group based on the experimental results, and stress–strain curves were plotted. Each experimental group included three parallel specimens, with results averaged.

#### 2.4.2. Wettability Test

The wettability of the electrospun nanofiber membrane surface was tested using an interfacial tension measuring instrument. Samples were cut into rectangular pieces measuring 50 × 10 mm^2^, adhered to a microscope slide, and placed on the workbench. Deionized water was used as the probe liquid, and the test method employed the static drop method. Continuous shooting mode recorded the changes in the water droplet at different time points. Each experimental group was repeated three times. Software was used to measure and calculate the water contact angle.

#### 2.4.3. Encapsulation Efficiency and Drug-Loading Capacity

Three sets of electrospun nanofiber membranes were prepared. They were extracted using a rotary stirrer in a PBS:trifluoroethanol = 1:10 mixed solution at 20 rpm. The extract was centrifuged at 10,000 rpm for 10 min at room temperature, before transferring supernatant to test tubes. Measurements were performed using a UV–visible spectrophotometer (UV-2102PC, Unico Instruments Co., Ltd., Shanghai, China) at λmax = 258 nm and λmax = 215 nm. To eliminate background interference, a PBS:trifluoroethanol = 1:10 mixture served as the control sample solution. Drug content in the ENPS was calculated using a calibration curve established under identical conditions.

$$EE\%=\frac{W_{e}}{W_{p}}\times 100\%$$

where *EE*% is the encapsulation efficiency, *W*_e_ is the actual measured drug amount in the nanofiber membrane, and *W*_p_ is the theoretical drug amount expected in the nanofiber membrane. All measurements were repeated three times.

$$DL\%=\frac{W_{e}}{W_{m}}\times 100\%$$

Here, *DL*% denotes the drug-loading efficiency, *W*_e_ represents the actual measured drug amount in the nanofiber membrane, and *W*_m_ denotes the mass of the nanofiber membrane. All measurements were repeated three times.

#### 2.4.4. In Vitro Dissolution Testing

The in vitro dissolution experiment was conducted according to the paddle method specified in the 2015 edition of the *Chinese Pharmacopoeia*. The SHZ-86 water bath constant-temperature shaker (Changzhou Jintan Shuibeike Pu Experimental Instrument Factory, Changzhou, China) was used for the in vitro dissolution test. Approximately 100 mg of the drug-loaded nanofiber membrane was immersed in 450 mL phosphate-buffered saline (PBS, pH 7.0, 0.1 M) at 37 °C with a rotation speed of 60 rpm. At predetermined time points, 4.0 mL of solution was extracted and replaced with 4.0 mL of fresh PBS. Absorbance was measured at λmax using a UV–visible spectrophotometer (UV2102PC, Unico Instruments Co., Ltd., Shanghai, China). The experiment was repeated six times. The cumulative drug release percentage (P%) of the product was calculated using the following formula:

$$P\%=\frac{c_{n}\times V_{o}+\sum_{i=1}^{n-1}c_{i}\times V}{Q_{o}}\times 100\%$$

where *V_o_* is the volume of dissolution medium (100 mL), *V* is the volume of extracted sample (4 mL), *Q*_o_ is the theoretical drug amount in each sample (mg), *c*_n_ is the drug concentration measured in the nth aliquot (mg/L), and *c_i_* is the drug concentration measured in the *i*-th aliquot (mg/L).

#### 2.4.5. CCK8 Cytotoxicity Assay

Three groups were established: Control, F3, and F6. The Control group received 1 mL/well of complete medium; the F3 and F6 groups received 8 mm diameter sample disks. Each treatment group included three replicate wells. L929 cells in the logarithmic growth phase were counted, adjusted to the desired concentration, and seeded at 5 × 10^4^ cells/well into a 24-well plate. The plates were incubated overnight at 37 °C in a 5% CO_2_ incubator to allow cells to adhere. Following the above grouping, the plates were incubated at 3 °C in a 5% CO_2_incubator for 12 h and 48 h. The medium was removed and wells were washed three times with PBS. A total of 1 mL/well of medium containing 10% CCK-8 was added. Incubation occurred at 37 °C in a 5% CO_2_ incubator for 2 h. Absorbance was measured at 450 nm using a microplate reader.

$$RCV\%=\frac{A_{s}-A_{b}}{A_{c}-A_{b}}\times 100\%$$

where *RCV*% represents relative cell viability, *A*_s_ is the absorbance value of the experimental group, *A_b_* is the absorbance value of the blank group (containing only CCK-8 reagent and medium), and *A_c_* is the absorbance value of the control group (normally cultured L929 cells). All measurements were repeated three times.

#### 2.4.6. Cell Live/Dead Staining Experiment

Three groups were established: Control, F3, and F6. The Control group received 1 mL/well of complete medium; the F3 and F6 groups received 10 mm diameter sample disks. L929 cells in the logarithmic growth phase were counted, adjusted to a concentration of 1.5 × 10^5^ cells/well, and seeded into confocal dishes. Cells were cultured overnight at 37 °C in a 5% CO_2_ incubator to achieve confluence. Following the above grouping, cultures were incubated for 12 h and 48 h. Reagent A (Calcein-AM) and reagent B (PI) were diluted 10-fold using dye dilution solution (Solution C). A total of 985.5 μL PBS was mixed with 10 μL diluted reagent A and 4.5 μL reagent B; these were prepared fresh for immediate use. Cells were washed once with PBS to remove excess serum. A total of 1 mL of staining solution was added per well and incubated at room temperature in the dark for 15 min. Staining was terminated by washing three times with PBS. Results were observed and images captured at 100× and 200× magnifications.

## 3. Results and Discussion

### 3.1. Electrospinning Process

**Figure 2:**
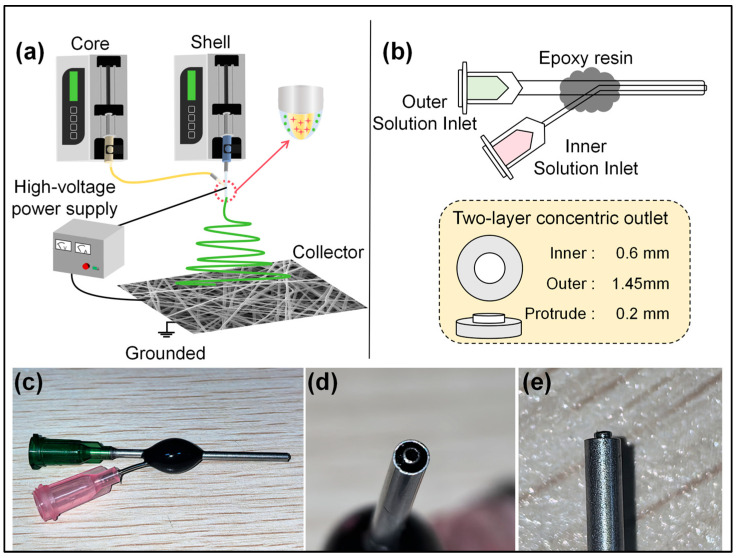
Coaxial electrospinning technique: (**a**) schematic of the coaxial electrospinning process; (**b**) internal structure of the spinneret; (**c**) physical image of the spinneret; (**d**) outlet view of the double-layer coaxial spinneret; (**e**) side view of the spinneret outlet.

Electrospinning technology, an efficient process for preparing nanofibers [54,55], utilizes a high-voltage electric field to stretch polymer solutions into ultrafine fibers ranging from tens of nanometers to several micrometers in diameter. These fibers are then collected to form fiber membranes with a high specific surface area and porous structures [56,57,58]. Electrospinning has evolved into complex configurations including coaxial [33,45,59,60,61], side-by-side [62,63], three-fluid coaxial, three-fluid side-by-side, and combined coaxial-side-by-side setups [49,64,65]. The coaxial electrospinning apparatus primarily consists of four components: an injection pump, a custom-made spinning nozzle, a grounded collector, and a high-voltage power supply [66,67]. Figure 2a illustrates a simplified schematic of the coaxial electrospinning process. The coaxial spinning nozzle is the key factor in forming core–shell nanofibers, making it the most critical component in the electrospinning process. This experiment utilized a custom-made spinning nozzle, as shown in Figure 2b. The two needles share a common axis. From the side view, the core-layer needle protrudes slightly by 0.2 cm, effectively guiding the leading fluid and delaying its potential diffusion. The connection is secured using epoxy resin. Detailed physical views of the spinning head can be found in Figure 2c–e, facilitating a clearer understanding of the structure.

**Figure 3:**
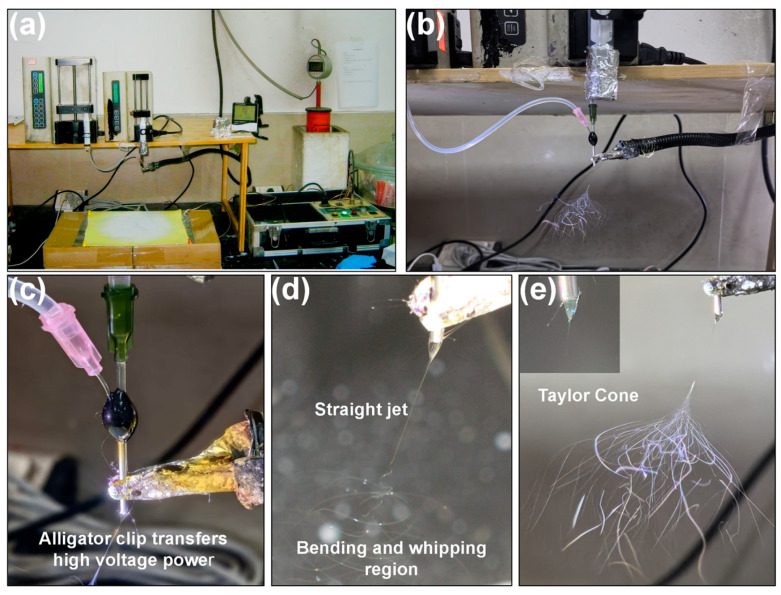
Coaxial electrospinning experimental process diagram: (**a**) schematic of spinning apparatus; (**b**) syringe connection to spinning head; (**c**) high-voltage power supply connection; (**d**) camera-captured solution stretching and solidification process; (**e**) smartphone-captured solution stretching and solidification process, with enlarged the Taylor cone view in upper left corner.

In this study, core–sheath nanofibers were prepared using a custom-made coaxial spinneret and a self-assembled electrospinning setup (Figure 3a). The number of syringe pumps and syringes primarily depends on the number of working fluids. Two syringe pumps were mainly used in this study, driving the core and sheath solutions, respectively. The syringes were connected to the spinneret via silicone tubing on one side and directly on the other (Figure 3b). The high-voltage power supply connected the positive electrode to the needle tip and the negative electrode to the collector, which was reliably grounded. The positive electrode primarily applied high voltage to the solution via the conductive properties of metal, secured to the spinneret head using an alligator clip (Figure 3c). Grounding the negative electrode ensured both equipment stability and operator safety while minimizing interference from high-frequency voltages during experiments.

Under the electrostatic spinning high-voltage electric field, the entire process from the Taylor cone tip to final deposition on the collector does not involve “pulling the solution into several segments at once.” Instead, it undergoes a continuous “multi-stage refinement” evolution. Laboratories often divide this into four distinct physical zones: the Taylor cone, the straight jet, the whipping and bending zone, and the solidified fiber region [68], as shown in Figure 3d,e. The upper right corner of Figure 3e displays an enlarged view of the Taylor cone. Methylene blue was incorporated into the inner PLA layer in this experiment, revealing a distinct core–sheath structure within the Taylor cone.

Although Figure 3d,e depict spinning processes at the same time interval, the whipping and bending region appears distinctly different between them. The image in 3d, captured by a camera, shows a continuous, curved whipping pattern, whereas the image in 3e, captured by a mobile phone, reveals a dispersed whipping pattern. This phenomenon primarily arises because the local draw rate during the whipping refinement stage of electrospinning reaches 10^3^–10^4^ s^−1^, equivalent to stretching a 1 cm liquid filament to 10 cm in 1 ms. This extreme rate is the core mechanism enabling electrospinning to directly form polymer into nanoscale fibers. The bending region appears “absorbed” in the smartphone footage not due to deformation but because the device’s low frame rate, progressive scanning, and insufficient lighting create temporal–spatial limitations that prevent the capture of millisecond-scale, nanoscale fiber movements. Consequently, only a “blurred thick line” is visible. High-speed cameras, however, employ global shutter technology at tens of thousands of frames per second combined with microsecond flashes to completely freeze micrometer-scale bending at a single instant. This allows for the observation of millisecond-scale, nanometer-scale, distortion-free, clear, and true-to-life bending details. This phenomenon also explains why the bending and whipping regions visible to the naked eye similarly appear as “illusionary” to the human eye, akin to the smartphone’s perception.

**Figure 4:**
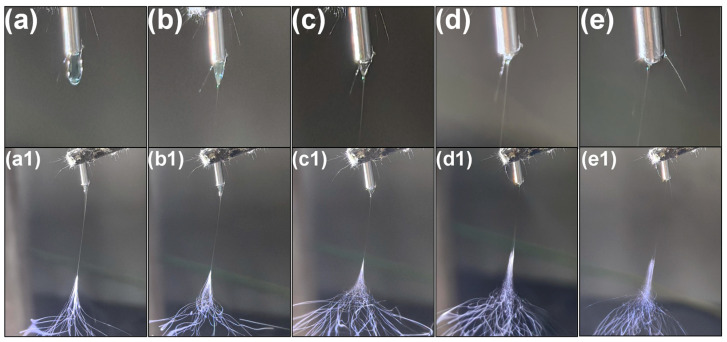
Effect of electrospinning process voltage on Taylor cones: (**a**) 4 kV; (**b**) 6 kV; (**c**) 8 kV; (**d**) 10 kV; (**e**) 12 kV; (**a1**–**e1**) correspond to the influence of different voltages on nanofibers during spinning.

Given the numerous parameters influencing electrospinning, Figure 4 primarily illustrates the effects of voltage on Taylor cone morphology and jet region characteristics during electrospinning. As voltage increases from 4 kV to 12 kV, observable changes in Taylor cone morphology emerge. At the lower voltage of 4 kV (Figure 4a,a1), the tip of the Taylor cone is relatively rounded, and the jet exhibits significant stretching, potentially leading to larger fiber diameters. As the voltage increases to 6 kV and 8 kV (Figure 4b,b1,c,c1), the tip of the Taylor cone becomes sharper, and the degree of jet stretching decreases, facilitating the formation of finer fibers. At 10 kV, the Taylor cone tip is extremely sharp, and jet stretching reaches its minimum, indicating this voltage is optimal for producing the finest fibers. However, when voltage is further increased to 12 kV, although the Taylor cone tip remains sharp, jet stability appears compromised, potentially leading to fiber formation irregularities. Therefore, increasing voltage can enhance fiber fineness, but an excessively high voltage may compromise fiber uniformity and quality, necessitating careful control during electrospinning. These observations are significant for optimizing electrospinning process parameters to obtain nanofiber materials with specific properties.

### 3.2. Morphology and Structure of Nanofibers

**Figure 5:**
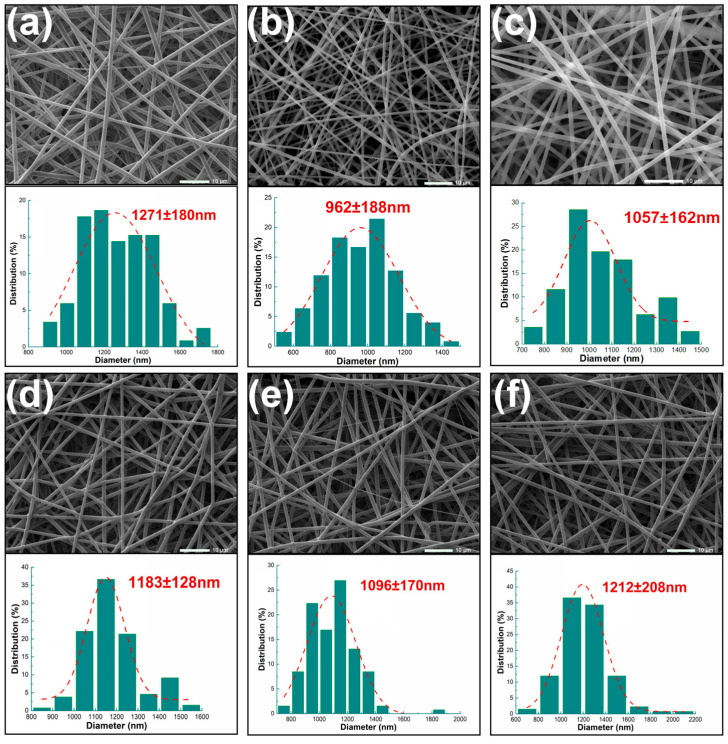
SEM images and diameter distribution of nanofiber membranes: (**a**) F1; (**b**) F2; (**c**) F3; (**d**) F4; (**e**) F5; (**f**) F6.

As shown in Figure 5, all six fiber groups exhibit continuous, non-beaded cylindrical structures, indicating that the selected solvent system and process parameters can yield stable electrospun nanofibers. Figure 5a,b show the surface morphology and average fiber diameter of nanofibers F1 and F2 prepared by single-fluid electrospinning. F1 fibers exhibit a slightly rough surface with an average diameter of 1271 ± 180 nm, the largest among the six samples. This may result from rapid moisture absorption causing phase separation and high expansion, leading to jet instability. F2 fibers exhibited the finest surface diameter of 962 ± 188 nm, with highly rigid PLA molecular chains and sufficient stretching, resulting in the smoothest surface consistent with its hydrophobic properties. Figure 5c–f show the surface morphology and average fiber diameter of the core–sheath nanofiber systems F3–F6. Their surface roughness falls between F1 and F2, with average diameters ranging from 962 ± 188 nm to 1271 ± 180 nm. The overall distribution exhibits a unimodal peak with relatively concentrated values. F3 exhibited an average fiber diameter of 1057 ± 162 nm, indicating that biphasic blending suppressed jet whipping and refined fiber structure. Upon adding small-molecule drugs, F4, F5, and F6 yielded average diameters of 1183 ± 128 nm, 1096 ± 170 nm, and 1212 ± 208 nm, respectively. The presence of drugs DPH and SH increased solution conductivity, further stretching the jet and slightly reducing fiber diameter in F1. However, due to drug incorporation, fiber diameters exhibited a minor increase relative to F3. The smaller increase in F5 may be attributed to enhanced solution viscosity and reduced whipping amplitude caused by the increased ionic strength of HBr in SH.

**Figure 6:**
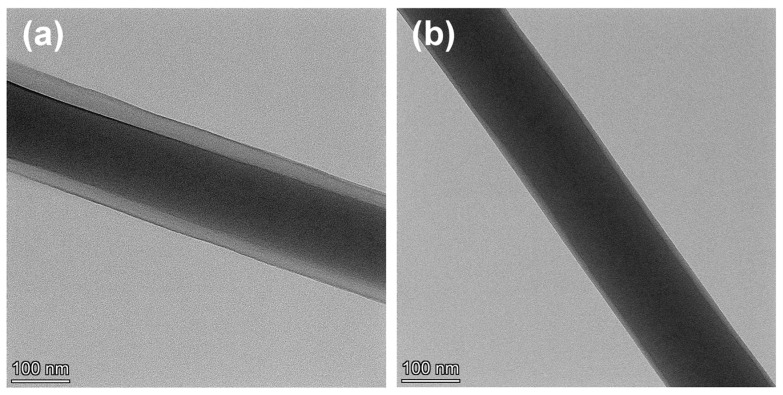
TEM images of nanofiber membranes: (**a**) F3; (**b**) F6.

Transmission electron microscopy (TEM) images of electrospun composite fibers visually reveal the regulatory effect of drug addition on fiber morphology and internal structure. TEM analysis of the internal structures of the drug-free core–sheath nanofiber F3 and the dual-drug core–sheath nanofiber F6 is shown in Figure 6. For the drug-free F3 fiber (Figure 6a), a distinct gray-scale difference is observed on both sides of the image, clearly indicating a pronounced core–sheath layer structure. In stark contrast, the F6 fiber incorporating DPH and SH (Figure 6b) exhibits a denser, more uniform surface structure with significantly reduced contrast. Nevertheless, the core–shell architecture remains discernible, indicating that drug molecules enhance the interfacial bonding between PVP and PLA. Notably, no distinct drug crystallization or particle agglomeration was observed within the F6 fiber, indicating that DPH and SH achieved uniform dispersion at the molecular or nanoscale within the composite fiber. This highly dispersed state not only prevents drug burst release but also provides a stable carrier structure for subsequent synergistic sustained release of the dual drugs.

### 3.3. Physical Properties and Compatibility of Nanofibers

**Figure 7:**
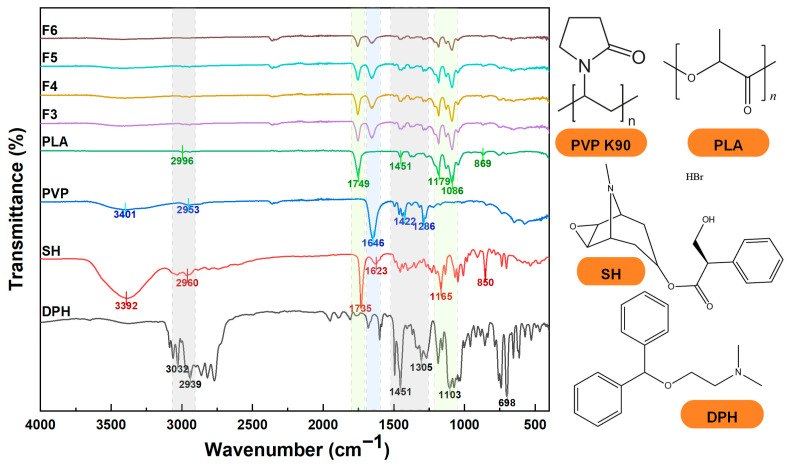
ATR-FTIR spectra and molecular formulas of raw materials (PVP, PLA, SH, DPH) and core–shell nanofibers F3–F6.

Figure 7 displays the structural formulas and ATR-FTIR spectra of raw materials including PVP, PLA, SH, and DPH, along with the ATR-FTIR spectra of core–sheath nanofibers F3–F6. By comparing the characteristic peaks of each component, all core–sheath nanofibers simultaneously retain the characteristic signals of PVP, PLA, and the drug, confirming the successful incorporation of the target drug into the nanofibers. Specifically, pure PVP K90 exhibits a characteristic peak at 1646 cm^−1^ for the pyrrolidone ring (C=O stretching vibration) and a broad, intense O–H stretching peak at 3401 cm^−1^ originating from adsorbed water and polymer chain-end hydroxyl groups. At 2953 cm^−1^ for alkyl C–H bonds in the main chain and pyrrolidone ring, along with CH_2_ bending at 1422 cm^−1^ and C–N stretching (amide) at 1286 cm^−1^, consistent with the literature values [69,70]. The characteristic absorptions of pure PLA are located at 1749 cm^−1^ (ester carbonyl C=O stretching), 2996 cm^−1^ (C–H stretching), 1451 cm^−1^ (CH_3_ asymmetric bending), 1179 and 1086 cm^−1^ (C–O–C, ester chain), and an 850 cm^−1^ noncrystalline region, consistent with the literature values [71,72]. DPH exhibits aromatic ring quadrupole stretching at 1602 and 1497 cm^−1^ aromatic ring stretching, with the ether bond C–O–C signal at 1244 cm^−1^ and protonated dimethylamino also exhibiting absorption in the 2700–2450 cm^−1^ region [73]. The characteristic peaks of SH crystals include 3392 cm^−1^ (amine NH stretching), 1623 cm^−1^ (aromatic C=C stretching), 850 cm^−1^ (aromatic C–H bending), 2960 cm^−1^ (alkyl C–H stretching), 1735 cm^−1^ (ester C=O stretching vibration), and 1165 cm^−1^ (ester C–O–C stretching vibration). The infrared spectrum also indicates a strong absorption band in the range of 3000–3700 cm^−1^. This very broad band indicates the presence of strong hydrogen bonds.

In sample F3, no other distinct peaks were observed besides those characteristic of PVP and PLA, indicating no drug was incorporated into this sample. In sample F4, alongside the characteristic peaks of PVP and PLA, the characteristic peak of DPH appeared at 3022 cm^−1^, 2960 cm^−1^, and 1650 cm^−1^, indicating the successful incorporation of DPH into the PVP sheath layer. In the F5 sample, besides the characteristic peaks of PVP and PLA, the characteristic peaks of SH also appeared, such as 2960 cm^−1^ and 1623 cm^−1^, indicating that SH was successfully introduced into the PLA core layer. Sample F6 exhibited characteristic peaks from PVP, PLA, DPH, and SH simultaneously, confirming the successful incorporation of both drugs into distinct phases. Additionally, the 1646 cm^−1^ peak of PVP in F6 showed a slight red shift, likely attributed to hydrogen bonding interactions between DPH and PVP. Overall, the sheath layer PVP stabilizes DPH via hydrogen bonding, while the core layer PLA fixes SH through hydrophobic interactions. This demonstrates that the core–sheath structure design fundamentally achieves the spatial separation and simultaneous loading of the two drugs, providing a structural foundation for subsequent programmed release studies and supporting the feasibility of electrospun fibers for dual-drug co-loading.

**Figure 8:**
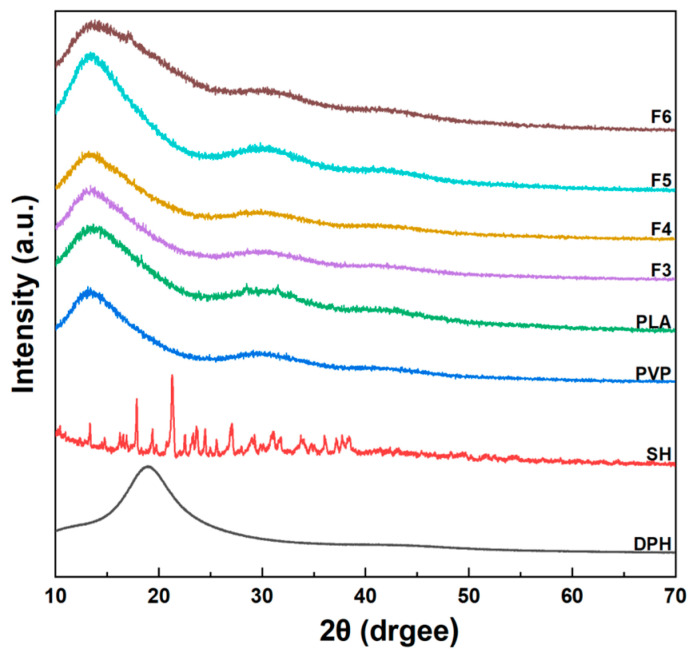
XRD analysis of raw materials including PVP, PLA, SH, and DPH, as well as core–sheath nanofibers F3–F6.

Figure 8 presents the XRD patterns of PVP, PLA, SH, DPH, and core–shell nanofibers F3–F6. PVP exhibits a broad amorphous halo centered around 20°, indicating its predominantly amorphous structure. PLA shows a broad peak around 16° and another broad peak centered around 28°, characteristic of its semi-crystalline nature with relatively low crystallinity. SH displays multiple sharp crystalline peaks in the range of 15–40°, indicating its high crystallinity. DPH shows a characteristic amorphous halo with no sharp diffraction peaks, confirming its amorphous nature in the raw material. For the core–shell nanofibers, F3–F6 all exhibit broad, diffuse diffraction patterns similar to those of PVP and PLA, with no sharp crystalline peaks observable. This indicates that the rapid electrospinning process not only reduces the crystallinity of PLA but also enables both DPH and SH to be uniformly dispersed in an amorphous state within the nanofiber matrices. Specifically, F4 shows only broad amorphous peaks without any characteristic crystalline peaks of DPH, suggesting DPH exists in an amorphous form within the fibers. F5 similarly displays only broad peaks with no SH crystalline peaks, confirming the successful incorporation of SH in an amorphous state. F6 exhibits broad amorphous characteristics without distinct crystalline peaks from either drug. These results demonstrate that the electrospinning process effectively transforms both crystalline (SH) and amorphous (DPH) drugs into amorphous solid dispersions (ASDs) within the polymer nanofibers. The rapid solvent evaporation during electrospinning prevents drug recrystallization, resulting in the molecular-level dispersion of the drugs within the polymer matrices. This amorphous solid dispersion formulation is advantageous for enhancing drug solubility and dissolution rates, thereby improving bioavailability.

### 3.4. Mechanical Testing

**Figure 9:**
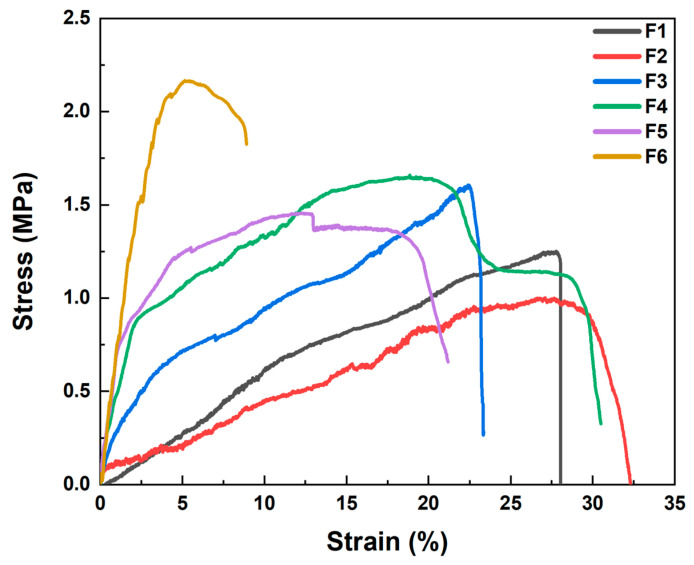
Stress–strain curves of nanofibers F1–F6.

Figure 9 shows the mechanical test results of six different nanofiber membranes, F1–F6, with each curve representing the stress–strain relationship of a membrane during loading. From the figure, it can be observed that F1 has a tensile strength of 1.23 MPa and an elongation at break of 27.93%, indicating high extensibility, which may suggest that PVP has good flexibility and ductility but relatively low tensile strength. F2 has a tensile strength of 1.00 MPa and an elongation at break of 27.10%, showing lower extensibility and a higher initial modulus, consistent with the rigidity and lower ductility of PLA. The curve of F3 lies between F1 and F2, with a tensile strength of 1.61 MPa and an elongation at break of 22.47%, indicating that its mechanical properties are a result of the mixture of PVP and PLA, offering moderate tensile strength and ductility. The tensile strengths of F4, F5, and F6 are 1.65 MPa, 1.46 MPa, and 2.17 MPa, respectively, and their elongations at break are 22.47%, 12.31%, and 5.16%, respectively. The curves show different mechanical behaviors, which may be due to the introduction of the drugs DPH and SH affecting the mechanical properties of the fiber membranes. In particular, F6, containing both drugs, may exhibit complex mechanical behavior, possibly related to the distribution and interaction of the drugs and their impact on the polymer matrix. These data indicate that the films have sufficient mechanical strength for handling and sublingual administration.

### 3.5. Wetting Performance Testing

**Figure 10:**
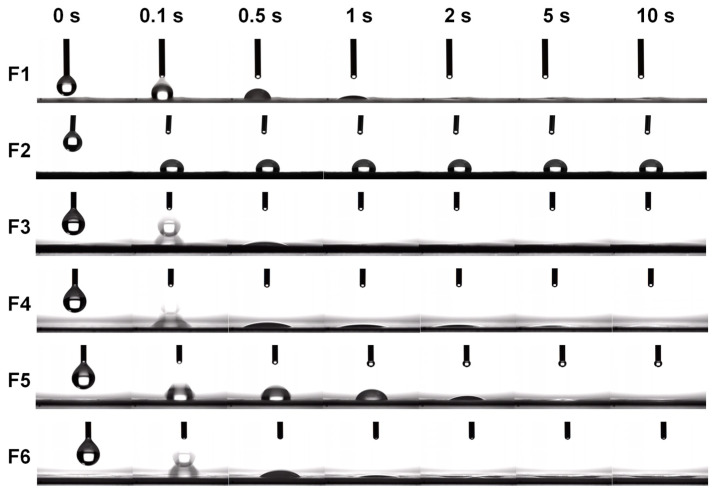
Dynamic changes in water droplets placed on F1–F6 nanofibrous membranes within 10 s.

Figure 10 details the dynamic changes in a water droplet placed on the surfaces of different nanofiber membranes F1–F6 over 10 s. F1 exhibits superhydrophilic properties, with the droplet fully spreading within 2 s after contact. At 0.5 s, the water contact angle reaches 69.4°, attributed to strong hydrogen bonding between the lactam groups in PVP molecules and water molecules. In contrast, F2 exhibits pronounced hydrophobicity, with the droplet maintaining a spherical shape throughout the 10 s period and a contact angle of approximately 105°, consistent with the inherent hydrophobic properties of PLA as a polyester polymer. F3 exhibits a unique rapid wetting behavior, with noticeable bubble dispersion occurring just 0.1 s after droplet contact, followed by rapid spreading. The water contact angle reaches 22.7° at 0.5 s and achieves complete wetting within 1 s. This phenomenon can be attributed to the hydrophilic microdomains formed by PVP within the PLA matrix acting as “water channels,” facilitating rapid water penetration. Concurrently, the selective dissolution of PVP leads to surface porosity, further enhancing wettability. Upon introducing hydrophobic DPH, the wetting rate of F4 significantly decreased, with a water contact angle of 32.2° at 0.5 s. The droplet exhibited slow spreading and formed a thicker liquid film. This indicates that the hydrophobic aromatic ring structure of DPH partially shielded the hydrophilic effect of PVP through hydrophobic interactions with PLA segments, thereby delaying water penetration. In contrast, F5 incorporating the hydrophilic drug SH maintained rapid initial wetting with a water contact angle of 78.6° at 0.5 s, but the droplet eventually stabilized in a partially spread state. This may result from electrostatic interactions between SH’s quaternary ammonium groups and PVP’s amide groups. In composite film F6, simultaneously loaded with DPH and SH, the water contact angle at 0.5 s was 39.8°, with wetting performance intermediate between F4 and F5. This indicates the material exhibits excellent surface wettability. Furthermore, by regulating the hydrophilic–hydrophobic balance of drugs within the PVP/PLA matrix, composite film materials with specific wetting behavior and release characteristics can be designed.

### 3.6. In Vitro Drug Release Performance Analysis

The encapsulation efficiency and drug-loading results showed that F4 had a drug-loading of 4.68% and an encapsulation efficiency of 93.09%, while F5 had a drug-loading of 2.88% and an encapsulation efficiency of 91.91%. Both samples exhibited high encapsulation efficiencies exceeding 90%, confirming that the core–shell structure formed by coaxial electrospinning effectively minimizes drug loss. PVP K90, as a hydrophilic polymer with excellent film-forming property [74,75], its combination as the shell layer with PLA as the core layer demonstrated excellent encapsulation compatibility for both anti-motion sickness drugs. The significant difference in drug-loading capacity is closely related to the drug binding sites and compatibility between the drug and the matrix. The superior compatibility between the PVP K90 sheath layer and DPH enables higher drug-loading capacity, while the polarity difference between the PLA core layer and SH limits the increase in drug-loading capacity.

**Figure 11:**
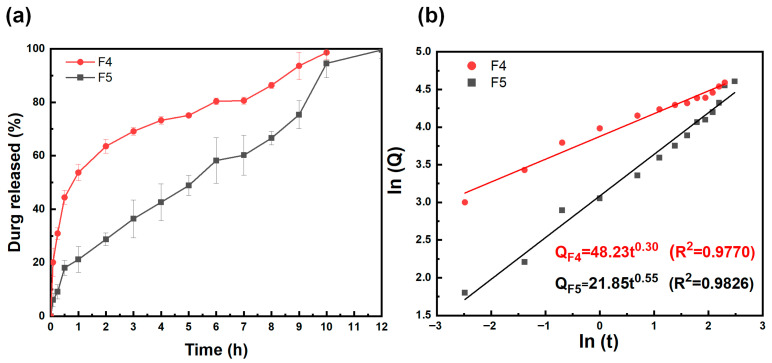
(**a**) In vitro drug release profiles of F4 and F5 drug-loaded nanofibers; (**b**) drug release mechanisms of F4 and F5 drug-loaded nanofibers.

Considering the core–sheath nanofiber structure, in vitro release behavior of F4 and F5 was analyzed in detail. Cumulative release profiles (Figure 11a) show F4 exhibits approximately 60% cumulative release within 2 h and approaches 100% at 10 h, demonstrating a biphasic “rapid onset + sustained release” profile. In contrast, F5 exhibits a more gradual release rate, approaching complete release only at 12 h, demonstrating a typical sustained-release profile. According to the Ritger–Peppas model fitting results (Figure 11b), F4’s diffusion index *n* = 0.30, significantly below 0.5, indicating that its release is primarily governed by Fickian diffusion. This behavior stems from F4’s drug distribution: DPH is dispersed in solution within the outer PVP layer. Upon contact with simulated saliva, the outer PVP rapidly swells to form hydrophilic channels, allowing drug molecules to diffuse rapidly along the concentration gradient. The hydrophobic barrier of the inner PLA layer only delays the release of a small amount of residual drug, without altering the overall diffusion-dominated mechanism. In contrast, F5 exhibits a diffusion index of *n* = 0.55, slightly greater than 0.5, consistent with non-Fickian diffusion (synergistic diffusion + carrier dissolution). This primarily stems from SH’s powder morphology: SH particles predominantly reside within the inner PLA layer. The channels formed by outer PVP swelling must penetrate the PLA matrix to access the drug, relying both on diffusion through PVP channels and the gradual erosion of PLA to expose drug particles. This synergistic effect results in a more sustained release profile.

The core–shell structure design further amplifies the impact of the drug on carrier interactions: In F4, the solution-state distribution of DPH synergizes with the rapid swelling of the outer PVP layer to achieve rapid onset, suitable for acute symptom relief in motion sickness. In F5, the particulate form of SH combines with the sustained-release properties of the inner PLA layer to achieve prolonged, steady release, making it better suited for the long-term prevention of motion sickness reactions. These results provide experimental evidence for achieving precise drug delivery with “rapid onset” and “long-lasting maintenance” by regulating the distribution morphology of drugs within core–shell structures.

### 3.7. CCK8-Based Cytotoxicity Assay

**Figure 12:**
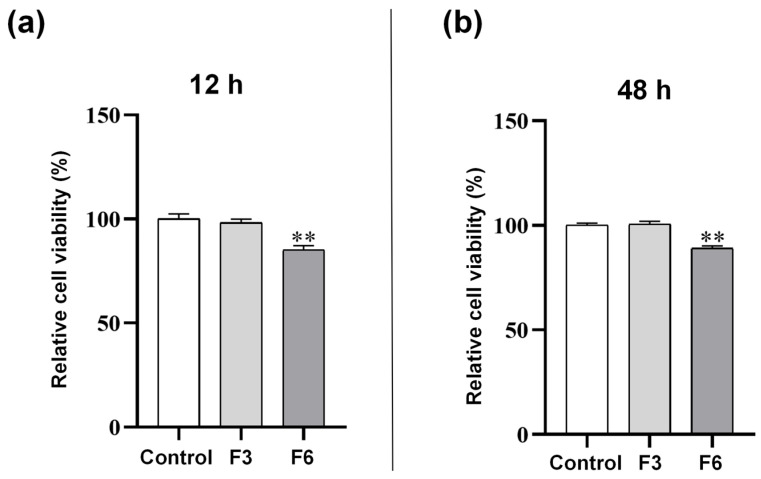
Changes in cell viability of L929 cells co-cultured with samples at 12 h and 48 h: (**a**) 12 h; (**b**) 48 h. ** *p* < 0.01.

Figure 12 shows the analysis of cell viability for the non-drug-loaded nanofiber F3 and the dual-drug-loaded nanofiber F6 using the CCK8 assay. In vitro cell culture confirmed the cellular compatibility of the oral drug-loaded nanofibers. The figure indicates that the relative cell viability of the F3 group showed no significant difference compared to the control group, demonstrating that this composite polymer carrier possesses good biocompatibility and does not exhibit significant toxicity to cells. In contrast, the F6 group co-loaded with DPH and SH maintained stable cell viability above 80% at both time points. The slight decrease compared to F3 primarily resulted from the combined pharmacological effects of diphenhydramine and scopolamine hydrobromide, rather than toxicity inherent to the carrier material itself. In the biomedical field, a cell viability ≥70% is commonly used as a reference threshold for assessing the biocompatibility of materials or drug delivery systems. Group F6 maintained cell viability above 80% after co-loading both drugs, significantly exceeding this critical threshold. Furthermore, a slight recovery was observed at 48 h compared to 12 h, further indicating that the cytotoxicity of this drug delivery system remains within a controlled and mild range, without inducing severe toxicity. This safety profile meets the basic requirements for subsequent in vivo experiments. This result demonstrates that the PLA/PVP carrier retains excellent biocompatibility after drug-loading. It also indicates that while both drugs exert their intended cell-inhibitory effects, they do not cause irreversible damage to cells, highlighting the design advantage of this drug delivery system: “effective drug release with low toxicity.”

### 3.8. Cell Live/Dead Staining Experiment Using Drug-Loaded Core-Sheath Nanofibers

**Figure 13:**
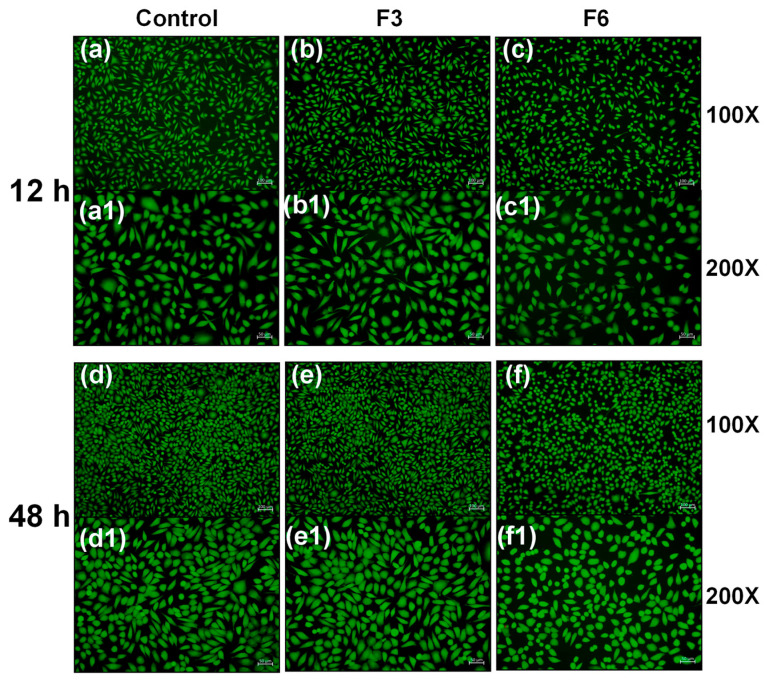
Cell viability of L929 cells co-cultured with samples at different time points: (**a**) 100× magnification: control sample co-cultured for 12 h; (**a1**) 200× magnification: control sample co-cultured for 12 h; (**b**) 100× magnification: F3 sample co-cultured for 12 h; (**b1**) L929 cells co-cultured with F3 sample for 12 h at 200× magnification; (**c**) co-cultured with F6 sample for 12 h at 100× magnification; (**c1**) L929 cells co-cultured with F6 sample for 12 h at 200× magnification; (**d**) co-cultured with control sample for 48 h at 100× magnification; (**d1**) co-cultured with control samples at 200× magnification for 48 h; (**e**) co-cultured with F3 samples at 100× magnification for 48 h; (**e1**) co-cultured with F3 samples at 200× magnification for 48 h; (**f**) co-cultured with F6 samples at 100× magnification for 48 h; (**f1**) co-cultured with F6 samples at 200× magnification for 48 h.

Figure 13 shows a cell viability analysis of unloaded nanofiber F3 and dual-drug-loaded nanofiber F6 via live/dead staining. Fluorescence microscopy observations are highly correlated with the quantitative viability data: the PLA/PVP polymer-only control group (F3, b, b1, e, e1) showed no significant differences in cell density or morphology at 12 h and 48 h compared to the control group (a, a1, d, d1), further confirming the excellent biocompatibility of the PLA/PVP carrier, which does not adversely affect cell adhesion and proliferation. In contrast, in the F6 group (c, c1, f, f1) co-loaded with DPH and SH, cell density at both time points was slightly lower than that of the control and F3 groups. However, cells maintained intact morphology and uniform green fluorescence, with no obvious signs of cell shrinkage or fluorescence quenching. This result visually reflects the mild inhibitory effect of the combined drugs on cell proliferation while indicating that this drug delivery system does not cause severe cellular damage during pharmacologic action. This finding corroborates the quantitative experiment showing F6 group cell viability maintained at approximately 80%, further validating the safety and efficacy of PLA/PVP as a combined drug delivery carrier.

## 4. Conclusions

This study successfully employed coaxial electrospinning technology to construct a core–sheath-structured nanofiber sublingual membrane with dual-drug release capabilities. PLA and PVP K90 served as composite carrier materials, loading diphenhydramine hydrochloride (DPH) and scopolamine hydrobromide (SH) as dual anti-motion sickness drugs. SEM and TEM images revealed the prepared nanofiber sublingual membrane exhibits’ regular linear morphology, with an average fiber diameter of 1212 ± 208 nm, forming a distinct and stable core–sheath nanostructure. XRD and FTIR spectra indicate that DPH is uniformly distributed in an amorphous state within the PVP K90 sheath layer, while SH is dispersed in an amorphous state within the PLA core layer. Wetting performance tests reveal that the wetting properties of this sublingual membrane lie between those of PLA monofiber membranes and PVP K90 monofiber membranes. In vitro drug release experiments clarified the release characteristics and mechanisms of both drugs: DPH exhibited a biphasic release profile of “rapid onset + sustained release,” with a cumulative release rate of 60% within 2 h and near-complete release by 10 h. The release mechanism was primarily Fickian diffusion (*n* = 0.30), enabling the rapid relief of acute motion sickness symptoms while maintaining sustained efficacy. SH exhibited a sustained-release profile, achieving near-complete release within 12 h. Its release mechanism involved non-Fickian diffusion (*n* = 0.55), enabling steady drug delivery after DPH onset to prolong overall efficacy duration and reduce dosing frequency. The synergistic and complementary release characteristics of both drugs effectively addressed the core challenges of slow onset and a short duration of action in traditional motion sickness medications. Cytotoxicity and live/dead staining experiments corroborate each other, confirming that cell viability remains above 80% after sublingual membrane application. This demonstrates the PLA/PVP K90 composite carrier’s excellent biocompatibility and safety profile. The efficacy and safety of the dual-antiemetic drug delivery system have been thoroughly validated. Furthermore, the sublingual film formulation eliminates the need for water during administration, significantly improving patient compliance and further highlighting the clinical advantages of this formulation. This study primarily focuses on in vitro characterization and biocompatibility research, resulting in certain limitations due to incomplete testing. Key experiments, such as accelerated stability testing and animal models for motion sickness validation, are lacking. Future work should optimize this system to provide more comprehensive data demonstrating the synergistic therapeutic efficacy of the dual-drug combination, thereby advancing the core–sheath fiber system toward clinical translation.

## Figures and Tables

**Table 1:** Experimental parameters for different electrospinning processes.

Sample	Electrospinning Technique	Fluid					Voltage (kV)	Receiving Distance (cm)	Product Morphology
		Sheath Layer	Drug	Core Layer	Drug	Flow Rate (mL/h)			
F1	Single-fluid electrospinning	PVP K90	/	/	/	1.0	7	15	Nanofiber
F2		/	/	PLA	/	0.8	8		Nanofiber
F3	Coaxial electrospinning	PVP K90	/	PLA	/	1.0/1.0	10		Nanofiber
F4		PVP K90	DPH	PLA	/	1.0/1.0			Nanofiber
F5		PVP K90	/	PLA	SH	1.0/1.0			Nanofiber
F6		PVP K90	DPH	PLA	SH	1.0/1.0			Nanofiber

## Data Availability

The data supporting the findings of this manuscript are available from the corresponding authors upon reasonable request.
